# The FOCCUS study: a prospective evaluation of the frequency, severity and treatable causes of gastrointestinal symptoms during and after chemotherapy

**DOI:** 10.1007/s00520-020-05610-x

**Published:** 2020-07-16

**Authors:** H. Jervoise N. Andreyev, Amyn Lalji, Kabir Mohammed, Ann C. G. Muls, David Watkins, Sheela Rao, Naureen Starling, Ian Chau, Sarah Cruse, Ville Pitkaaho, Jennifer Matthews, Laura Caley, Victoria Pittordou, Carolyn Adams, Linda Wedlake

**Affiliations:** 1 The Department of Gastroenterology, Lincoln County Hospital, United Lincolnshire Hospitals Trust, Greetwell Rd, Lincoln, LN2 5PT UK; 2 The Biomedical Research Centre, Nottingham Digestive Diseases Centre, The School of Medicine, University of Nottingham, Nottingham, UK; 3 The Department of Medicine, The Royal Marsden NHS Foundation Trust, Sutton, London, UK; 4 Clinical Research and Development Department, The Royal Marsden NHS Foundation Trust, Sutton, London, UK; 5 Royal Marsden Clinical Trials Unit Royal Marsden NHS Foundation Trust, Sutton, UK

**Keywords:** Cancer, Chemotherapy, Gastrointestinal, Side effects, Toxicity, Quality of life, Malabsorption, Bile acid, Disaccharide, SIBO, Small intestinal bacterial overgrowth, Surgery, Radiotherapy, Endoscopy, Diarrhoea, Abdominal pain, Bloating, Incontinence

## Abstract

**Background:**

The underlying mechanisms of chemotherapy-induced gastrointestinal (GI) symptoms are poorly researched. This study characterised the nature, frequency, severity and treatable causes for GI symptoms prospectively in patients undergoing chemotherapy for GI malignancy.

**Methods:**

Patients receiving chemotherapy for a GI malignancy were assessed pre-chemotherapy, then monthly for 1 year using the Gastrointestinal Symptom Rating Scale, a validated patient-reported outcome measure. Patients with new, troublesome GI symptoms were offered investigations to diagnose the cause(s). Their oncologist was alerted when investigations were abnormal.

**Results:**

A total of 241 patients, 60% male, median age 63 years (range 30–88), were enrolled; 122 patients were withdrawn, 93%, because of progressive disease or death. During the study, > 20% patients reported chronic faecal incontinence and > 10% reported moderate or severe problems with taste, dysphagia, belching, heartburn, early satiety, appetite, nausea, abdominal cramps, peri-rectal pain, rectal flatulence, borborygmi, urgency of defecation or tenesmus. Thirty percent reported continuing passage of hard stools and 30% on-going diarrhoea. Moderate or severe fatigue affected 40% participants at its peak and persisted in 15% at 1 year. Toxicity dictated change in chemotherapy for 13–29% patients/month. Common Terminology Criteria for Adverse Events underestimated gastrointestinal morbidity. Pre-chemotherapy screening identified previously undiagnosed pathology: exocrine pancreatic insufficiency (9%), vitamin B_12_ deficiency (12%) and thyroid dysfunction (20%). Patients often refused investigations to diagnose their chemotherapy-induced symptoms; however, for every three investigations performed, one treatable cause was diagnosed: particularly small intestinal bacterial overgrowth (54%), bile acid malabsorption (43%), previously not described after chemotherapy, and unsuspected urinary tract infection (17%).

**Conclusions:**

Patients undergoing chemotherapy for GI malignancy commonly have difficult GI symptoms requiring active management which does not occur routinely. The underlying causes for these symptoms are often treatable or curable. Randomised trials are urgently needed to show whether timely investigation and treatment of symptoms improve quality of life and survival.

**Trial registration:**

ClinicalTrials.gov Identifier: NCT02121626

**Electronic supplementary material:**

The online version of this article (10.1007/s00520-020-05610-x) contains supplementary material, which is available to authorized users.

## Introduction

In 2015, 359,960 people in the UK were newly diagnosed with cancer. Mortality rates have fallen over the last 10 years for most cancers, for example, oesophageal cancer by 7%, bowel cancer 15% and 32% for gastric cancer [1]. Research continues to develop better therapies.

However, now that effective treatments for many forms of cancer exist, it has become increasingly important to maintain and improve quality of life after diagnosis rather than focussing solely on reducing cancer mortality. To improve or maintain quality of life often means treating the symptoms caused by the cancer or the anti-cancer therapies. To do this effectively may require the development of new services providing expert supportive care which would not only improve patient well-being but also enhance delivery of the optimal cancer treatment, uncompromised by toxicity [2].

GI symptoms are common in patients with cancer. They may predate the cancer or arise because of the tumour or de novo as a side effect of treatment. It is not unusual for GI symptoms to impinge on patients’ quality of life and to interfere with cancer treatment prompting dose reduction or cessation of therapy potentially resulting in less effective tumour control. When abnormal GI symptoms persist long term, they can have a devastating impact [3].

Some GI symptoms are well managed. For example, severe vomiting once prevented delivery of effective but extremely emetogenic chemotherapeutic regimens. Intensive research produced potent anti-emetic drugs. Consequently, vomiting is rarely a significant problem for patients with cancer. Much less attention has been paid to the incidence, severity or optimal management of other GI symptoms commonly reported by patients such as anorexia, altered taste, borborygmi, bloating, constipation, diarrhoea, dysphagia, early satiety, frequency of defaecation, incontinence, mucus discharge, nausea, nocturnal defaecation, pain/cramps, reflux, regurgitation, steatorrhoea, tenesmus, urgency of defaecation, weight loss and wind.

It is widely believed that GI symptoms arise as a result of pathological changes caused by chemotherapy. However, the evidence to support this hypothesis is weak. In the upper GI tract, chemotherapy causes mucositis characterised by increased apoptosis and pathological changes such as decreased villous height. The intensity of chemotherapy correlates with the severity of the histological change [4]. However, the severity of the symptoms patients develop with similar degrees of mucositis can vary widely. Evidence from non-malignant disorders also suggests that histological change correlates poorly with symptoms [5].

**Fig. 1:**
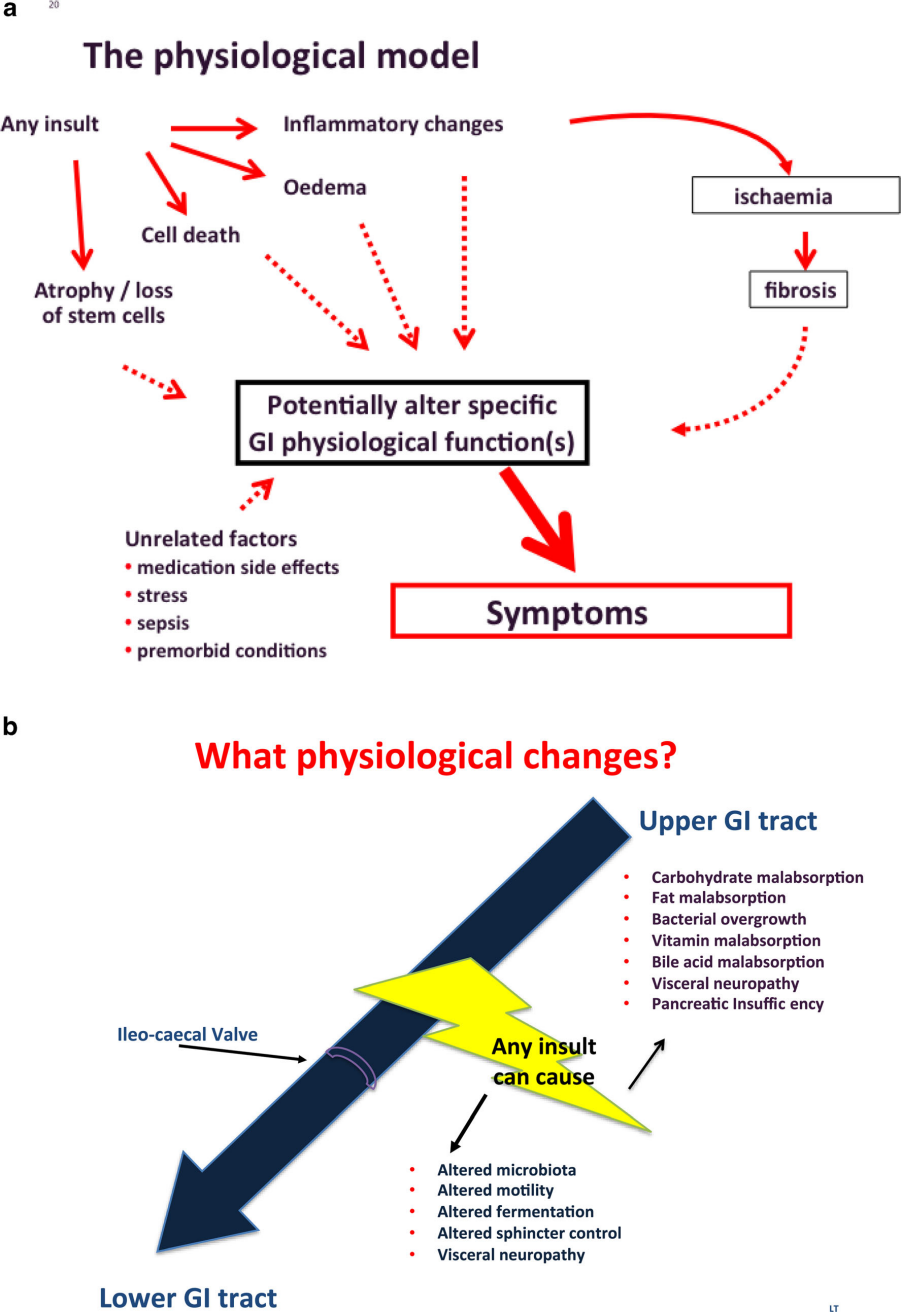
**a** The physiological base for GI symptoms. **b** What type of physiological changes

Instead, we have proposed an alternative model for the development of GI symptoms (Fig. 1a, b) namely, that abnormal GI symptoms developing as a result of cancer therapies are caused primarily by disrupted GI physiological function(s). Physiological upset may be triggered by different factors including pathological changes. The differing physiological reserves between individuals explain why patients with the same degree of pathological change do not always develop the same symptoms. We tested this hypothesis in a large randomised trial in patients with new-onset GI symptoms developing after pelvic radiotherapy [6]. In that study, we demonstrated that if the clinician focused on identifying which abnormal physiological processes had developed, treatable abnormalities—e.g. bile acid malabsorption, carbohydrate malabsorption, exocrine pancreatic insufficiency, small intestinal bacterial overgrowth (SIBO) and vitamin deficiency—were frequently detected. In addition, we showed that in the majority of patients, several different physiological abnormalities developed at the same time. However, clinical judgement predicted poorly which physiological abnormalities were present [7]. Instead, if clinicians arranged specific investigations for each symptom, using checklists for accuracy, correct diagnoses could be made rapidly, appropriate treatments prescribed and significant clinical benefit achieved at a relatively low cost [6, 8]. Other studies support these findings [9–12]. Indeed, a nurse can be trained to assess and manage the large majority of such patients by following such checklists no less effectively than a gastroenterologist [6]. This approach seems to be valid in patients treated with a wide variety of therapies for cancer not just radiotherapy [8].

In patients with GI cancers, which comprise more than 20% of all new cancer diagnoses, chemotherapy is an integral part of treatment for many, often in combination with surgery and/or radiotherapy and/or biological therapies. A number of treatable physiological abnormalities have been described following surgery, radiotherapy and treatment by anti-cancer biological agents [8, 10, 11], but there has been virtually no research to identify the physiological mechanisms which lead to similar symptoms in patients receiving chemotherapy.

The FOCCUS (Focussing on Cancer Chemotherapy’s Undiagnosed Symptoms) study was carried out to quantify prospectively the incidence, severity, frequency and impact on quality of life of chemotherapy-induced GI symptoms. It aimed to document how frequently treatable causes for those symptoms could be identified.

## Methods

This was a prospective, observational, single-centre cohort study in patients aged 18 years or above undergoing chemotherapy for GI malignancies in the GI unit of one specialist cancer hospital. The study was reviewed and approved by the institutional clinical research committee and by the appropriate ethics committee. The study was conducted in accordance with the Helsinki Declaration, 1975 (revised 1983). Patients gave written informed consent before study enrolment.

### Patients

Patients with an histologically proven GI malignancy were eligible to participate once they were scheduled for chemotherapy. Patients participating in studies with conflicting end points were excluded. Disease progression was a pre-specified criterion for withdrawal of the patient from the study.

Data was collected at baseline (pre-treatment) and monthly for 1 year after start of chemotherapy. However, to avoid additional appointments, study follow-up visits coincided with chemotherapy delivery cycles.

Participants were asked to provide several pre-chemotherapy samples over and above standard practice including blood tests for thyroid function and vitamin B_12_ levels and stool for pancreatic faecal elastase. Urine was dipped at each study visit and if it showed abnormalities potentially suggestive of infection, was sent for culture.

At the initial baseline and subsequent visits, patients were assessed for the presence of GI symptoms by completing a modified Gastrointestinal Symptom Rating Scale (GSRS) questionnaire [13]. This questionnaire asked patients to rate each of 25 bowel symptoms as occurring “never”, “occasionally”, “frequently affecting” or “causing major change” to their life. Previous studies have shown that symptoms in the first two categories can be categorised as “mild” while symptoms in the second two categories can be categorised as “moderate/severe”. In this paper, we only report symptoms in the moderate/severe categories apart from faecal incontinence where we report any occurring. Patients were also asked to complete a question on tiredness and frequency of defecation (Supplemental figure 1), and a Bristol stool chart was used to help patients explain the types of stool they passed [14]. Participants also completed at each study visit a St. Mark’s Faecal Incontinence score [15] and a FACT-G version 4 quality-of-life questionnaire [16]. The researcher, after directly questioning the patient, graded symptoms using the Common Terminology Criteria for Adverse Events (CTCAE) version 4 [17].

If patients indicated on their GSRS questionnaire new-onset moderate/severe symptoms which were not present before the start of chemotherapy, peer-reviewed checklists were used to determine which investigations they required to diagnose the cause for those symptoms [18, 19]. If these investigations were acceptable to the patient, they were booked by study personnel.

The study personnel were research nurses or research dieticians. We have previously shown that non-medical personnel can be trained to investigate abnormal GI symptoms in people after chemotherapy as effectively as a doctor, if detailed checklists for symptoms diagnosis are employed [6].

If investigations were abnormal, the treating oncologist was informed and provided—if necessary—with a brief protocol to help them manage potentially unfamiliar conditions. Telephone access to the Chief Investigator (a gastroenterologist) was offered if required to help with management. Study personnel were trained in the use of the checklists, and their skills were updated at monthly meetings with the Chief Investigator. The checklists were refined and simplified at regular meetings throughout the study (Appendix 1 in the Electronic supplementary material [ESM]: final version).

### Statistics and data analysis

The primary end point of this observational study was the frequency of GI symptoms. Secondary end points included the range and severity of GI symptoms, the number of investigations recommended and performed to diagnose the cause for symptoms and their results. Study data were summarised using descriptive analysis methods for continuous data, using mean and standard deviation or median and range/inter-quartile range as appropriate, and for categorical data using counts and percentages. Before the study opened, we aimed to recruit as many patients as possible over 2 years, and follow each patient for 12 months. There was no a priori power calculation to justify the numbers, and as a result, no group comparisons were planned. We calculated, however, based on historic referral rates that the maximum possible sample size would be 500 patients.

## Results

### Patient demographics

**Fig. 2:**
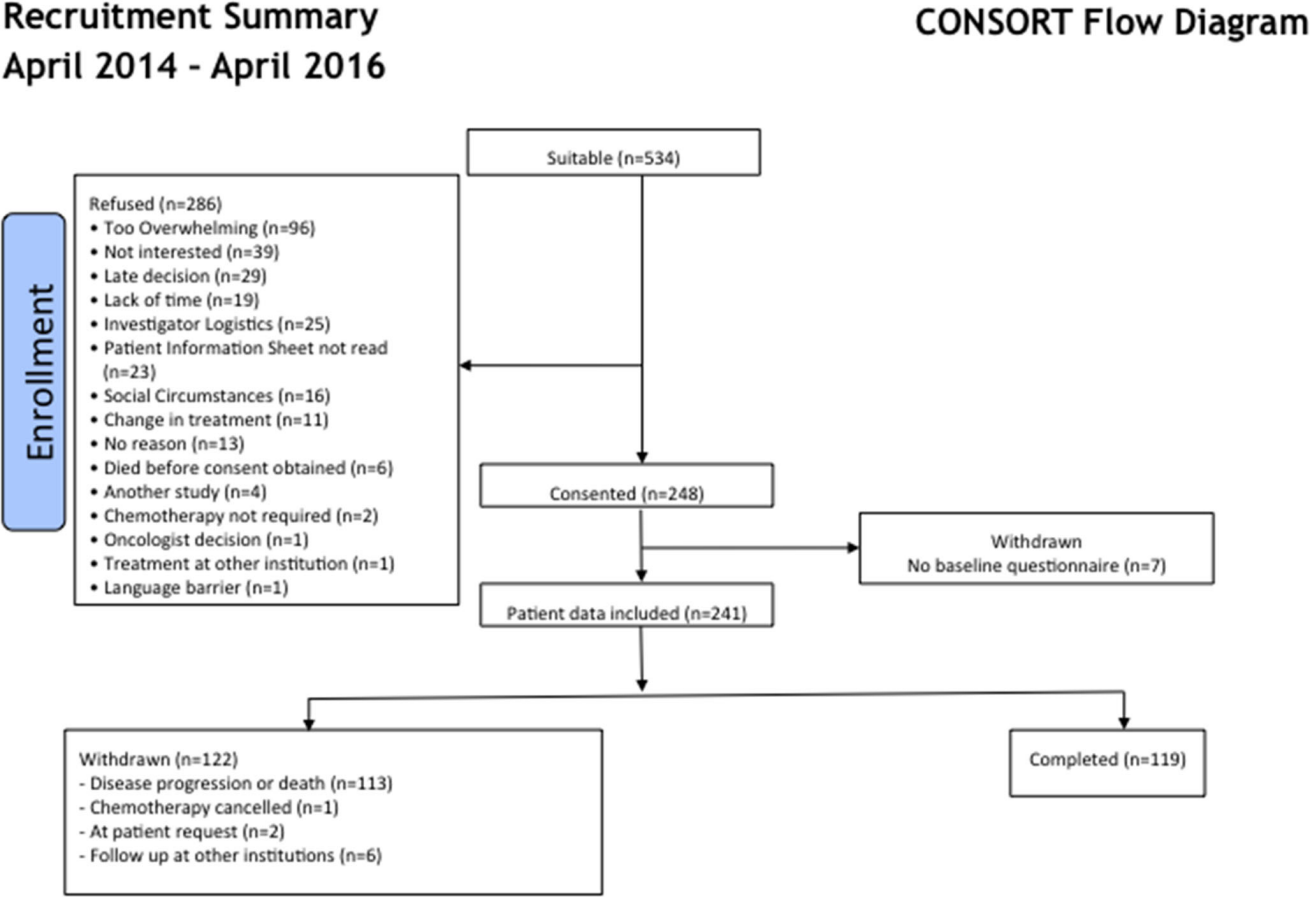
The consort diagram showing the recruitment of patients into the study

Recruitment started in April 2014; follow-up was completed in June 2017. A total of 248 patients, 150 (60%) males with a median age of 63 years (range 30–88), consented to take part (Fig. 2). Patients’ demographics are summarised in Table 1. Seven patients did not provide a baseline symptom questionnaire so were excluded from further analysis. Of the 241 patients included, 183 provided questionnaires up to 3 months, 157 to 6 months and 119 patients completed a 12-month questionnaire. Withdrawals from the study were because of disease progression or death (*n* = 113, 89% cancer-related, 11% unrelated causes), followed up arranged at institutions other than our own (*n* = 6), at patients’ request (*n* = 2) and because their chemotherapy was cancelled (*n* = 1).

**Table 1:** Patient characteristics

Variables	Male, *n* = 150	Female, *n* = 98	Total, *n* = 248
Median age in years (range)	64 (32–88)	62 (30–87)	63 (30–88)
Primary cancer site, *n* = (%)			
Anal	2 (1)	6 (6)	8 (3)
Colon	35 (23)	29 (30)	64 (26)
Hepatobiliary	8 (5)	8 (8)	16 (6)
Oesophagus/OGJ	46 (31)	21 (22)	67 (27)
Pancreas	16 (11)	9 (9)	25 (10)
Rectum	26 (17)	14 (14)	40 (16)
Small intestine	0	1 (1)	1 (0.4)
Stomach	9 (6)	5 (5)	14 (6)
Other	4 (3)	1 (1)	5 (2)
Unknown primary	4 (3)	4 (4)	8 (3)
Previous GI cancer-related surgery			
Yes	65 (43)	46 (47)	111 (45)
Numbers with a stoma			
Colostomy	8 (47)	3 (37)	11 (44)
Ileostomy	9 (53)	5 (63)	14 (56)
Non-GI comorbidities:			
Cardiac	28 (19)	13 (13)	41 (17)
Diabetes mellitus	16 (11)	7 (7)	23 (9)
Other endocrine	20 (13)	12 (12)	32 (13)
Hypertension	38 (25)	24 (24)	32 (13)
Melanoma	3 (2)	1 (1)	1 (0)
Prostate cancer	13 (9)	0	13 (5)
Psychiatric	0	1 (1)	1 (0)
Respiratory	15 (10)	12 (12)	27 (11)
Rheumatological	8 (5)	10 (10)	18 (7)
Vascular disease	0	2 (2)	2 (1)
Other	33 (22)	19 (19)	52
Treatment pathway			
Chemotherapy alone	74 (49)	50 (51)	124 (50)
Chemotherapy and radiotherapy	28 (19)	20 (20)	48 (19)
Surgery and chemotherapy	36 (24)	23 (23)	59 (24)
Surgery, chemotherapy and radiotherapy	12 (8)	5 (5)	17 (7)

GI cancer surgery undertaken before chemotherapy included colonic resection (*n* = 38), anterior resection (*n* = 17), stoma formation (*n* = 25), Whipple resection (*n* = 4) and gastric bypass/resection (*n* = 3). Two patients had had a gastro-duodenal stent placed. One patient had active ulcerative colitis. Twenty-six patients had undergone radiotherapy. Participants’ tumour stage, other comorbidities and chemotherapy regimens prescribed are shown in Appendix 2 in the ESM.

Supplemental figure 2 shows during each month of the study the percentages of patients receiving chemotherapy, stopping at least one chemotherapy drug or undergoing a chemotherapy dose reduction. A median 16.5% patients (range 12.5–28.9%) each month required change of chemotherapy treatment for toxicity. We did not record how much of this altered treatment was due solely to GI toxicity.

### GI symptoms

**Fig. 3:**
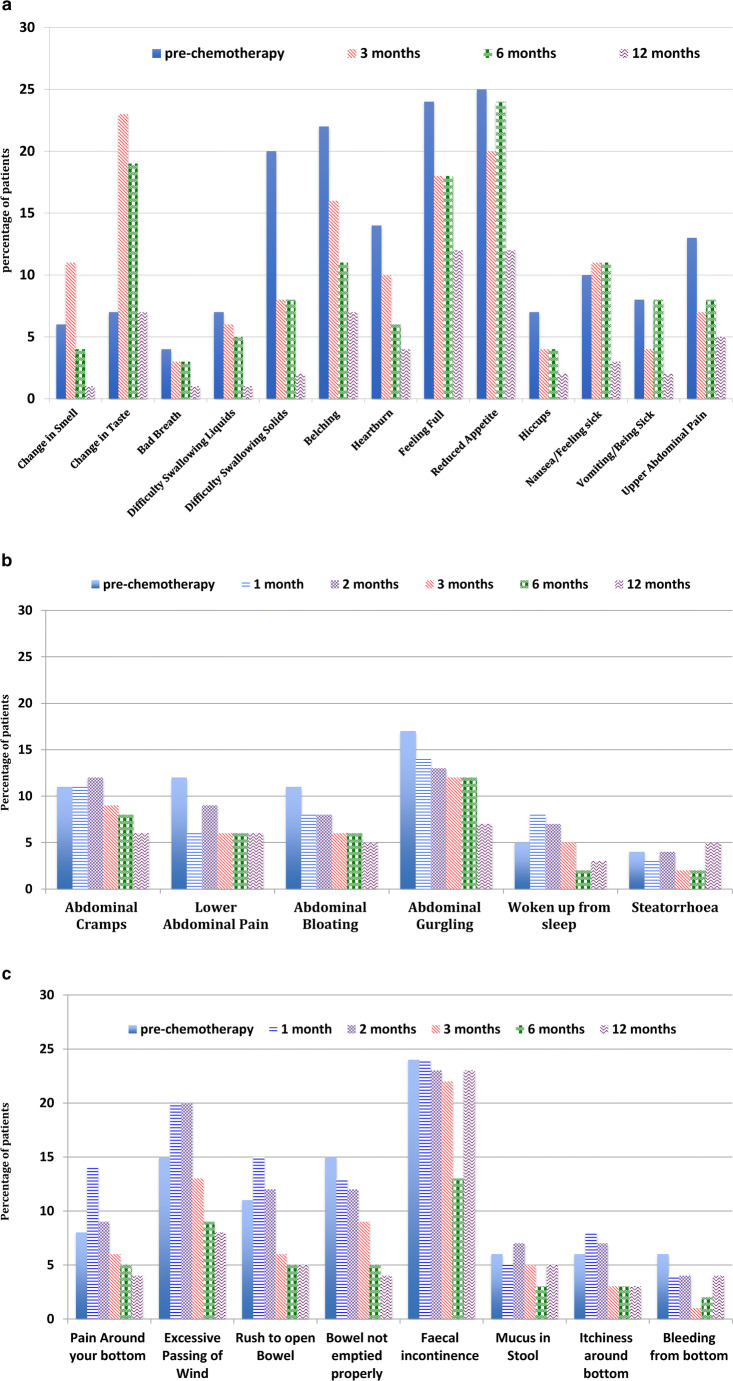
**a** Percentage of patients reporting moderate or severe upper GI symptoms. **b** Percentage of patient reporting moderate to severe abdominal symptoms. **c** Percentage of patients reporting moderate to severe lower GI symptoms or any faecal incontinence

The number of patients reporting moderate/severe GI symptoms or any faecal incontinence during the study is shown in Fig. 3a, b and c. Some symptoms were reported less frequently with chemotherapy: dysphagia for liquids and solids, belching, heartburn, early satiety, upper abdominal pain and tenesmus. The frequency of some symptoms increased after the onset of chemotherapy: change in smell, change in taste, peri-rectal pain, rectal flatulence and urgency of defaecation. Some symptoms changed in reported frequency very little in the first 6 months but were less frequent at a year: bad breath, reduced appetite, bloating, hiccoughs, nausea, vomiting and borborygmi. The frequency of some symptoms was largely unchanged throughout the study: faecal incontinence, rectal mucus discharge, peri-anal itching, lower abdominal pain/cramps, rectal bleeding, the need for nocturnal defecation and steatorrhoea. Three of these symptoms, worsening rectal mucus discharge, nocturnal defaecation and steatorrhoea, suggest organic disorders which are potentially curable. However, it should be possible to offer therapeutic options to improve many of the other symptoms [18, 19].

Approximately, half of all patients had intermittent type 6 and 7 stools (i.e. diarrhoea) or intermittent type 1 or 2 (hard or very hard) stools. Over time, bowel frequency became more normal (normal bowel frequency is defined as defecation occurring between 3 times a day and once every 3 days) but one in 5 patients fell outside this normal range in the first 6 months (Supplemental figures 3 and 4a&b).

The GSRS questionnaires suggested that approximately 1 in 5 patients reported faecal incontinence; this did not change much month by month. The data obtained using the sensitive St. Mark’s Incontinence score (Supplemental figure 5) was consistent with the GSRS data.

Moderate/severe lethargy affected 26% patients pre-chemotherapy and was reported by 40%, 40%, 36%, 35% and 15% at 1, 2, 3, 6 and 12 months respectively. Body mass index changed very little for the whole cohort through the course of the study with a mean of 25.92 (standard deviation 4.98) pre-chemotherapy and 26.65 (5.43) at 12 months.

Patients’ symptoms were graded using the CTCAE v4 for every comparable category of GI symptom, frequently recorded less severe toxicity (data not shown) than was demonstrated by the GSRS. For example, CTCAE scoring assessed 1–9% fewer patients as suffering from faecal incontinence, a distressing symptom, at comparable stages.

The quality-of-life results are shown in Supplemental figure 6. A high score is better than a low score. A total score can be calculated by adding the individual physical, emotional, social and functional subscale scores. Social and emotional well-being are very important to quality of life and are unlikely to change as quickly as the scales which measure function and physical health. Physical and functional scores appear to improve at 12 months when fewer people were receiving chemotherapy although the standard deviation is wide. It is questionable how representative these results are when half of all the patients were withdrawn from the study mostly because of progressive disease.

### Causes for abnormal symptoms

This study set out not only to document the frequency with which symptoms were occurring but also to determine the underlying causes for symptoms.

Pre-chemotherapy, 27 out of 196 (14%) patients had a low baseline vitamin B_12_ level, 38 out of 188 (20%) patients had a TSH (0–78) outside the normal range, 17 out of 196 (9%) patients had pancreatic faecal elastase levels which were abnormal and 9 of 65 (14%) mid-stream urine (MSU) samples sent indicated urinary infection.

If patients developed new GI symptoms, pre-specified investigations for each symptom were offered to patients. Additional blood tests were almost always accepted. Of these, during follow-up, 75 patients had their vitamin B_12_ rechecked, and in 25 (35%), it had become abnormal. Forty-eight patients underwent thyroid function tests and the TSH was outside the normal range in 15 (31%). After abnormal urinary analysis, 387 MSU samples were sent for culture, of which 67 (17%) showed unequivocal infection.

For other tests, uptake was poor (Table 2). At 334 follow-up appointments, breath testing was recommended, mostly as a diagnostic test for SIBO but in 11% of patients to exclude carbohydrate malabsorption. Only 53 of these tests were performed; 21 patients of 39 (54%) who underwent glucose hydrogen methane breath testing had a result suggestive of SIBO. The other tests were performed to exclude mono- or disaccharidase malabsorption for either fructose, lactose, or sucrose: one (7%) was suggestive of fructose malabsorption. The number of endoscopies recommended/taken up is shown in Table 2 and Supplemental figure 7. The abnormalities reported from the few procedures performed were high (51%). Knowledge of the abnormal findings—oesophagitis (25%), hiatus hernia (25%), gastritis (25%), duodenitis (15%), gastric polyp (5%) and radiation proctopathy (5%)—may have allowed better targeted symptomatic treatment.

**Table 2:** Number (%) of major investigations recommended and the number taken up at each follow-up during the study

	Pre-chemotherapy	Month 1	Month 2	Month 3	Month 4	Month 5	Month 6	Month 7	Month 8	Month 9	Month 10	Month 11	Month 12
Endoscopy recommended	9	50	45	52	42	38	28	22	17	24	16	24	1
Endoscopies performed	1 (11)	9 (18)	5 (11)	3 (6)	3 (7)	2 (5)	2 (7)	3 (13)	1 (6)	1 (4)	3 (18)	5 (21)	0
Breath test recommended	11	49	41	48	37	42	27	20	17	22	16	24	1
Breath tests performed	2 (18)	9 (18)	7 (17)	2 (4)	7 (19)	3 (7)	1 (3)	5 (25)	1 (6)	5 (23)	5 (31)	8 (33)	0
SeHCAT scans recommended	4	20	17	23	20	23	19	14	10	9	8	13	1
SeHCAT scans performed	1 (25)	3 (15)	4 (24)	3 (13)	2 (10)	4 (17)	1 (5)	4 (28)	0	2 (22)	2 (25)	4 (31)	0

SIBO [20] and carbohydrate malabsorption [21] are previously reported complications of chemotherapy; bile acid malabsorption has not been previously described as a cause for chemotherapy-induced diarrhoea. During the FOCCUS study, a ^75^selenium homocholic acid taurine acid (SeHCAT) scan, the definitive diagnostic test for bile acid malabsorption, was offered to 217 patients, performed in 30 and abnormal in 13 (43%) (Table 2 and Supplemental figure 8a&b). Abnormal scans were found in most tumour groups, 1 abnormal scan out of 4 patients assessed after treatment for oesophageal cancer, 2 abnormal out of 5 treated for pancreatic cancer, 1 abnormal of 3 treated for hepatobiliary cancer, 4 abnormal of 7 treated with colonic cancer, 1 abnormal of 4 treated for rectal cancer, 2 out of 2 with peritoneal primaries and 1 abnormal out of 3 treated for an unknown primary. These patients were treated with a variety of chemotherapies including capecitabine single agent, capecitabine with oxaliplatin, gemecitabine and one patient epirubicin, cisplatin and 5-fluorouracil.

## Discussion

This is the first study assessing the frequency of life-changing GI symptoms in patients receiving cancer chemotherapy. We show that standard oncological toxicity scoring commonly used in oncology underestimated GI morbidity. Chemotherapy improved symptoms which could be attributed to a cancer causing partial obstruction to the GI lumen but many other symptoms worsened. Significant numbers reported unremitting symptoms.

No previous study has investigated systematically why patients have these difficult symptoms. When routine gastroenterological investigative tests were used, one in 5 patients had treatable but missed comorbidities before chemotherapy, particularly thyroid dysfunction, vitamin B_12_ deficiency and exocrine pancreatic insufficiency. During and after chemotherapy, frequently identified causes which are curable with antibiotics include small intestinal bacterial overgrowth in one in every two patients with suggestive symptoms and unsuspected urinary tract infection in 3 in 10 patients with abnormal urine analysis. In other patients’ symptoms, e.g. those with bile acid malabsorption (4 in 10 patients with loose stool), treatments either completely cure or significantly improve symptoms [8].

While this was a heterogeneous population, the mechanisms causing GI symptoms appeared to be similar across tumour types. Our findings are consistent with animal studies, case reports, small series and even data from patients with non-malignant GI disorders which suggest that inflammatory processes predispose to loss of function by the barrier layer of the gut and that specific biochemical functions of receptors and enzyme symptoms in the GI tract are altered temporarily or permanently [21, 22]. This leads to exactly the same types of physiological dysfunction of the GI tract as we report in patients after treatment with radiotherapy [23].

Many clinicians believe that chemotherapy causes predictable, usually mild GI symptoms, and that investigations are unnecessary as patients improve adequately when given symptomatic treatments. This perception may partly result from the inadequacy of the questionnaires measuring toxicity used in clinical practice which are often not comprehensive and fail to reflect accurately the patient experience [24–26]. There has been little focus by clinicians on the symptoms which patients consider particularly difficult [27]. Possibly, this is because even specialists with an interest in the GI tract frequently do not understand which symptoms matter to the patient and the extent to which these symptoms affect quality of life [28]. This is why a rapid shift to the routine use of patient-reported outcome measures (PROMs) is important for clinicians and patients alike: PROMs increase the frequency of appropriate discussion during consultations and are associated with improved symptom control, better supportive care and patient satisfaction [29].

It is frequently stated that clinical experience is adequate at identifying symptoms which require investigation and those which can be treated empirically. However, we increasingly understand that this approach is naïve in patients with GI benign disorders [30] let alone those symptoms which develop after complex anti-cancer therapies, where often multiple causes contribute. The treatable GI diagnoses found in this study, bile acid malabsorption, carbohydrate malabsorption, exocrine pancreatic insufficiency and SIBO, produce similar symptoms (Table 3) and frequently coexist. Even expert clinicians struggle to identify the correct diagnoses for such symptoms from the history alone [7].

**Table 3:** The possible GI symptoms caused by different physiological disorders

Symptoms:				
	Bile acid malabsorption	Carbohydrate malabsorption	Exocrine pancreatic insufficiency	Small intestinal bacterial overgrowth
Abdominal pain/cramps		X	X	X
Belching				X
Bloating	X	X	X	X
Borborygmi		X	X	X
Constipation				X
Flatulence	X	X	X	X
Frequency	X	X	X	X
Foul smelling stool			X	X
Incontinence	X	X	X	X
Loose stool	X	X	X	X
Rectal mucus discharge				X
Nausea/vomiting				X
Nocturnal defaecation	X	X	X	X
Steatorrhoea	X		X	X
Urgency	X	X	X	X
Weight loss	X		X	X
Weight gain	X			

When so many patients had on-going difficult symptoms, it is puzzling why the majority declined the investigations offered. Various reasons may have contributed. It is widely believed that difficult GI symptoms are inevitable during chemotherapy. For decades, the priority has been to develop effective treatments for cancer with almost no research into improved symptom management unless that symptom (e.g. neutropaenia or intractable vomiting) threatened delivery of chemotherapy. Patients are offered symptomatic treatments and are told that things will rapidly improve once chemotherapy is completed. Possibly, patients were preoccupied with getting their cancer treated and feared that our tests might delay/stop their treatment. Or else, patients who do not feel well and are already spending a lot of time attending the hospital may become reluctant to undergo “research” tests which they feel are unlikely to benefit them and which were proposed by researchers, not their treating clinician. It was not unusual for patients to postpone recommended investigation(s) until the symptoms had persisted for several months. This is understandable for endoscopic investigations which are invasive, unpleasant and probably undertaken in this patient group a number of times already. However, this is less understandable for a breath test or a SeHCAT scan which is not onerous, carries minimal risk, is not unpleasant and has such potential to reduce symptom burden by diagnosing treatable conditions [6, 8].

This study was not designed to show the benefit to the patient from diagnosing treatable disorders. Nor were the research team conducting the study trained to deliver the treatments required to address the abnormalities found. After arranging investigations, we informed the patient, oncology team and the GP of what we had found and recommended treatment, but did not record whether those treatments were given or whether they were given in the way we recommended.

If people with cancer present with bowel dysfunction before treatment, they are more likely to suffer severe bowel toxicity from treatment [26]. We know that targeted, early supportive care significantly improves outcomes [31, 32] [32] and when symptom control is integrated with the provision of oncological treatment, this improves quality of life [33]. It is therefore an intuitive assumption that better gastroenterological supportive care will improve difficult GI symptoms and quality of life for patients and diminish the requirement to consider chemotherapy dose reduction. Between one in eight and one in three patients, each month required their initially prescribed regimen to be changed because of toxicity. While we are unable to determine how many of these changes were due to GI toxicity alone, our study suggests that potentially many patients are having their chemotherapy intensity reduced potentially unnecessarily if more effort was devoted to diagnosing and treating the underlying cause of GI toxicity adequately. However, it will require a randomised trial to determine whether survival is enhanced by timely gastroenterological supportive care.

Our approach in this study was not radical; it followed long-established principles of good general medical practice. Our findings suggest that a much more robust and holistic assessment of patients at presentation to the oncology unit should be routine. Urgent consideration needs to be given how best to manage the large numbers of patients with difficult bowel function quickly. Probably, this requires a nurse practitioner trained in gastroenterology with rapid access to the relevant diagnostic facilities to be embedded in the oncology clinic.

## Conclusion

The FOCCUS study demonstrates that difficult bowel symptoms before, during and after chemotherapy are common and for the first time has demonstrated that there are frequently treatable causes for these symptoms. A randomised controlled trial is required to demonstrate the size of the survival benefit and the impact on quality of life by offering these patients’ expert gastroenterological supportive care.

## Electronic supplementary material

ESM 1 (DOC 331 kb)

ESM 2 (DOCX 237 kb)
