# Supplementary material for: The FOCCUS study: a prospective evaluation of the frequency, severity and treatable causes of gastrointestinal symptoms during and after chemotherapy
Source: Support Care Cancer. 2020 Jul 16;29(3):1443–53. doi: 10.1007/s00520-020-05610-x (PMC7843552; doi:10.1007/s00520-020-05610-x)
Supplement: Supplementary file 1 — (DOC 331 kb) [file 520_2020_5610_MOESM1_ESM.doc]

**FOCCUS Study (CCR 3921)**

**Investigational algorithm for new onset**

**GI symptoms in GI patients during chemotherapy.**

**Version 13**

## Introduction

This guide is designed to mainly aid clinical nurse specialists and research nurses looking after gastrointestinal (GI) cancer patients undergoing chemotherapy with gastrointestinal symptoms working in conjunction with a gastroenterologist

This Guide defines best practice although not every investigation modality or treatment will be available in every trust.

Those using the Guide, especially if non-medically qualified, should identify a senior gastroenterologist or other appropriately qualified and experienced professional whom they can approach easily for advice if they are practicing in an unsupervised clinic.

Practitioners should not use this guide outside the scope of their competency and must identify from whom they will seek advice about abnormal test results which they do not fully understand before using the Guide.

Specific therapies are usually not listed by name but as a “class” of potential drugs as different clinicians may have local constraints or preferences as to the medications available.

Important principles to consider when using this algorithm are:

The patient may have multiple symptoms

Often, patients have more than one diagnosis simultaneously

Each symptom may have more than one cause

Symptoms must be investigated systematically otherwise causes may be missed

Arranging all investigations indicated by the algorithm at the first consultation reduces follow-up and allows directed treatment at all causes for symptoms at the earliest opportunity

This Guide has two parts:

1. Introduction, how to use the algorithm, taking a history, abbreviations and guide to blood tests.
2. An algorithm detailing the individual investigations and treatment of each of the symptoms.

## How to use the algorithm

1. Identify the symptoms by systematic history taking
2. Examine the patient appropriately
3. Use the algorithm to plan investigations
4. For symptoms present at baseline wait 6 weeks to see if symptom(s) improve with treatment prior to booking algorithm investigations
5. For patients undergoing radiotherapy: if lower GI cancer sites wait 6 weeks after competing radiotherapy prior to booking lower GI investigations. For upper GI cancer sites wait 6 weeks after competing radiotherapy prior to booking upper GI investigations
6. Most patients have more than one symptom and investigations need to be requested for each symptom
7. Usually all investigations are requested at the same time and the patient reviewed with all the results
8. Treatment options are generally offered sequentially but this should be discussed with oncology team and they should use their clinical judgment

## Abbreviations used in the algorithm

5-HIAA 5-Hydroxyindoleacetic acid

CNS Clinical Nurse Specialist

CT Computer Tomography

ECG Echocardiogram

EPI Exocrine Pancreatic Insufficiency

ERCP Endoscopic retrograde cholangio-pancreatogram

EUS Endoscopic Ultrasound

GI Gastro Intestinal

GORD Gastro-oesophageal reflux disease

ICP Intracranial pressure

MRI Magnetic Resonance imaging

NET Neuro endocrine Tumour

OGD Upper endoscopy (oesopago-gastroduodenoscopy)

PET Positron Emission Tomography

SIBO Small intestinal Bacterial Overgrowth

US Ultrasound

## Taking an appropriate history

Patients cannot be helped without an accurate history being taken.

- Taking a history of GI symptoms is a skill which must be learnt.
- Tools such as a Bristol Stool Chart can often clarify exactly what patients mean.
- Specialist units find that symptom questionnaires completed by the patient before the consultation often helps clarify which issues are really troubling the patient.

**Taking a history needs to elicit:**

- What was bowel function like before the cancer emerged?
- How have the symptoms changed over time?
- Are key features indicative of reversible underlying pathology present, e.g.
  - Steatorrhoea?
  - Nocturnal waking to defaecate?
  - Rapid progressive worsening of symptoms?
  - Rapid weight loss?
  - Has the patient noticed any masses?
- Patients and clinicians alike often miss the presence of intermittent steatorrhoea – ask:
  - Is there an oily film in the lavatory water?
  - Is the stool ever pale / putty-like / foul smelling / difficult to flush/ floaty?
- A very clear definition of what a patient means when they use specific terms –
- e.g. “diarrhoea” – what type on the Bristol Stool Chart?; “frequency” – true bowel
- opening or tenesmus and incomplete evacuation?
- Is there a consistent impact of a specific component of diet on their symptoms, especially:
  - Fibre: how much are they eating – too much / too little?
  - Fat: does this promote loose stool / steatorrhoea?
  - Lactose-containing foods?
  - Gluten-containing foods?
  - Alcohol intake?
- Is there an association between the start of specific medication or increase in its dose and their symptoms – e.g. metformin, lansoprazole, beta blockers?

## Guideline for blood tests used within the Guide

| Haemoglobin <125 g/l | Inform oncology immediately |
| --- | --- |
| Abnormal urea, Electrolytes | - If sodium <120 or >150 this is an emergency inform oncology immediately  -If Potassium >5 or <3.5 inform oncology team immediately |
| Abnormal liver function test | – Inform oncology team immediately |
| Abnormal glucose level | **If no history of diabetes:**  >11mmol/L inform oncology team, this is an emergency  **If known diabetic:**  >11 mmol/L, handover to oncology team within 24 hours. |
| Abnormal calcium level | - If 2.6–2.9mmol/L: discuss with supervising clinician within 24 hours.  - If >3.0mmol/L: **this is an emergency**. Inform Oncology team immediately |
| Elevated CRP | -Inform clinicians |
| RBC folate deficiency | – Inform oncology team. |
| Iron deficiency: ferritin, % transferrin saturation, Red cell indices | –Inform oncology team. |
| Low Vit B12 | Discuss with clinician: |
| Abnormal thyroid function tests | -– If TSH suppressed (<0.5mIU/L), recheck result with thyroid auto antibodies.  – If TSH suppression confirmed, discuss with clinician to organise radiological imaging and treatment.  – If TSH elevated (>4.0mIU/L). Recheck result. Also check morning cortisol and synacthen test if Na ≤135mmol/l/ K >4mmol/l or raised urea or creatinine.  – If TSH elevation confirmed: Discuss with clinician to start thyroid replacement  medication. Request GP monitor long-term. Review bowel function after 6–8 weeks. |
| Abnormal coeliac serology | -Inform oncology team and recommend referral to gastroenterology. |
| Mg | If <0.3mmol/l this is an emergency.  Notify clinician within half an hour  – If 0.3–0.5, Inform oncology team. |

## GI symptoms

**Symptom page**

Belching 7

Bleeding (rectal) 8

Bloating/abdominal cramps. 9

Borborygmi. 11

Constpation/difficulty evacuating rectum 12

Change in smell 13

Change in taste 14

Diarrhoea 15

Dysphagia 18

Early satiety 19

Faecal incontinence 20

Flatulence (rectal) 21

Frequency of defecation (see diarrhoea) 15

Halitosis 22

Hiccups 23

Jaundice 25

Loss of sensation 26

Mucus discharge 27

Nausea and vomiting 28

Nocturnal defecation (see diarrhoea) 15

Pain (upper abdominal/epigastric) 30

Pain (lower abdominal) 32

Pain (anal/perianal/rectal) related to defeacation 34

Pain (anal/perianal/rectal) not related to defeacation 35

Pruritis (perianal) 36

Reduced appetite 37

Reflux (Acid / Bile) / heartburn 38

Regurgitation 39

Steatorrhoea 40

Tenesmus 41

Urgency of defaecation (see diarrhoea) 15

Weight loss (unintentional) 42

## Belching / burping

The release of gas from the digestive tract, mainly the oesophagus and stomach through the mouth

| **Investigations** | **Potential results** |
| --- | --- |
| Fibre quiz and assess if high fizzy drink intake | Excessive use of carbonated drinks/ inappropriate fibre intake |
| Medications assessment | Use of effervescent medications |
|  | Metformin |
| OGD + D2 aspirate and glucose hydrogen methane breath test  NB OGD not recommended until 6 weeks post-upper GI surgery for testing for SIBO | SIBO |

## Bleeding (rectal)

| **Symptoms / Toxicity** | **Grade 0** | **Grade 1** | **Grade 2** | **Grade 3** |
| --- | --- | --- | --- | --- |
| **Bleeding** | None | Blood post passing stool when wiping | Blood staining underwear. | - Blood in toilet bowl   Frank haemmorraghe |
| **Action** | None | - Inform the oncology team  - Follow the algorithm | - Flexisig  - Inform the oncology team within 24h | Inform the oncology team immediately |

**NB If patient rectal/anal cancer below investigations not appropriate, inform oncology team.**

| **Investigations** | **Potential results** |
| --- | --- |
| **If bright bleeding** |  |
| First line | Inform oncology team  Flexi-sig (if NAD go to second line)  NB flexi sigmoidoscopy not recommended until 6 weeks post-lower GI surgery |
| Second line | Colonoscopy  NB Colonoscopy not recommended until 6 weeks post-lower GI surgery |
| **If dark bleeding** |  |
| First line | OGD and colonoscopy (If NAD go to second line)  NB Colonoscopy not recommended until 6 weeks post-lower GI surgery and OGD not recommended until 6 weeks post-upper GI surgery for testing for SIBO |
| Second line | Discuss with chief investigator capsule endoscopy |

## Bloating

| **Symptoms / Toxicity** | **Grade 0** | **Grade 1** | **Grade 2** | **Grade 3** |
| --- | --- | --- | --- | --- |
| **Abdominal Distension / Bloating**  Bowel obstruction? Ascities?  Constipation? Anorexia? – (see specific toxictity) | None | No other symptoms  Not troublesome | Symptomatic but not interfering with GI or resp fn. | - Symptomatic and severly altered GI / resp fn. - Life threatening consequences, eg operative intervention |
| **Action** | None | Monitor | Follow the Algorithm | Emergency  Inform the oncology team immediately |

| **Investigations** | **Potential results** |
| --- | --- |
| **❖ First line** |  |
| Blood screen  (U&Es, LFT, CRP, RBC Folate, B12, glucose) | Abnormal results.  Minimally raised CRP, raised RBC Folate and reduced B12 could be suggestive of SIBO |
| Abdominal Xray  NB only do if currently experience symptom  (if had CT of abdomen in past month ask doctor to review this instead) | Severe faecal loading  Bowel obstruction  (review other symptoms if indicates faecal loading, such as nocturnal defecation, incontinence, increased bowel frequency with normal stool type, urgency of defecation) |
| Diet assessment (Fibre and sorbitol quiz)  Medications assessment | Inadequate / excessive fluid or fibre intake  Excessive sorbitol |
| OGD + duodenal aspirate and biopsy  Glucose hydrogen methane breath test  NB OGD not recommended until 6 weeks post-upper GI surgery for testing for SIBO | SIBO |
| Faecal elastase | Faecal elastase |
| **❖ Second line** |  |
| If previously positive for SIBO and still symptomatic re-do OGD + duodenal aspirate and glucose hydrogen methane breath test  NB OGD not recommended until 6 weeks post-upper GI surgery for testing for SIBO | SIBO |
| **❖ Third line** |  |
| Lactose breath test | Lactose breath test |
| **❖ Fourth line** |  |
| Fructose Breath Test | Fructose Breath Test |
| **❖ Fifth line** |  |
| Sucrose Breath Test | Sucrose intolerance / malabsorption |
| **❖ Sixth line** |  |
| Ask oncology team to consider  Abdominal ultrasound / CT scan  (if no previous imaging in last 6 months) | Suggestive of gallstones, inflammatory bowel disease, tumour recurrence, other |

## Borborygmi

A rumbling / gurgling noise produced by the movement of fluid or gas through the intestine

| **Investigations** | | **Potential results** |
| --- | --- | --- |
| **❖ First line** |  | |
| Blood test (CRP, RBC Folate, B12) | | Abnormal results |
| OGD + duodenal aspirate and biopsies ± glucose hydrogen methane breath test  NB OGD not recommended until 6 weeks post-upper GI surgery for testing for SIBO | | SIBO |
|  |  | Enteric infection |
| **❖ Second line** |  | |
| If previously positive for SIBO and still symptomatic re-do OGD + duodenal aspirate and glucose hydrogen methane breath test  NB OGD not recommended until 6 weeks post-upper GI surgery for testing for SIBO | | SIBO |
| **❖ Third line** | |  |
| Lactose Breath Test | | Lactose intolerance/ malabsorption |
| **❖ Fourth line** | |  |
| Fructose Breath Test | | Fructose intolerance /malabsorption |
| **❖ Fifth line** | |  |
| Sucrose Breath Test | | Sucrose intolerance / malabsorption |

## Constipation / difficulty evacuating rectum

**If bowels not opened, increased distension, no flatus with nausea ± vomiting, This is an emergency inform oncology team immediately**

| **Investigations** | **Potential results** |
| --- | --- |
| **❖ First line** |  |
| Dietary / lifestyle/ medications assessment | Inadequate fibre intake  Reduced general exercise  Drug induced e.g.  – opioid  – ondansetron  – anti-muscarinic  – loperamide  – iron supplement  - Verapamil  Chronic constipation /evacuation disorder (not to book investigations if patient has this) |
| Flexible sigmoidoscopy  For longstanding problems (>1 week/recurrent) | Anal Fissure  Newly diagnosed IBD  Anastomotic structuring |
| Blood test (U&Es, electrolytes, Calcium, Albumin) | Dehydration |
| **❖ Second line** |  |
| Abdominal X-Ray  NB only do if currently experience symptom  NB do not do if patient has ilestomy  (if had month ask doctor to review this instead) | Faecal loading / Faecal impaction |
| **❖ Third line** |  |
| Transit study | Slow GI Transit |

## Change in smell

| **Investigations** | **Potential results** |
| --- | --- |
| Symptom assessment | if impacting patient significantly inform oncology team. |

## Change in taste

| **Investigations** | **Potential results** |
| --- | --- |
| **❖ First line** |  |
| Medication assessment | Chemotherapy induced |
|  | Medication induced  Metronidazole |
| Visual inspection of mouth | Oral candida |
|  | Dental problems |
| **❖ Second line** |  |
| Post- Chemo ask the oncology team to consider  CT/ MRI/ PET head | Base of skull disease |

## Diarrhoea (stool type 6-7 Bristol Stool Chart)

Also use this section if patient has ‘Frequency of defecation (bowels opening ≥4 times a day or increased from baseline)’,

‘Nocturnal defecation’ or ‘Urgency of defecation’

NB for ilestomy please see ilestomy section

For colostomy patient’s type 6 stool is normal and not for algorithm investigations for this. For colostomy patients must assess frequency of defecation by measuring output in jug over 24 hours prior to booking investigations. For colostomy patients type 4 or 5 stool is normal.

| **Symptoms / Toxicity** | **Grade 0** | **Grade 1** | **Grade 2** | **Grade 3** |
| --- | --- | --- | --- | --- |
| **Diarrhoea**  **Consider infection!**  Duration?How many days has this occurred for?  How many times in a 24hr period?  Does the patient have any abdominal pain/discomfort?  For how long? Has the patient taken any medication?  See specific toxicity for pain | None | Increase to 2-3 bowel movements a day over pre-treatment movements  Drink more fluids  Obtain stool sample  ? consider regimen specific antidiarrhoeal | Increase 4-6 episodes a day or nocturnal movement/moderate cramping  Drink plenty of fluids  Obtain stool sample  ? consider regimen specific antidiarrhoeal | Increase to 7-9 episodes a day or incontinence  Severe cramping  Increase to >10 episodes a day or grossly bloody diarrhoea or need for parenteral support |
| **Action** | None | Monitor | Follow the Algorithm | Emergency  Inform the oncology team immediately |

If grade 0-1 for <7 days monitor. If grade 2 or above inform oncology team and CNS. If symptoms ≥7 days or recurrent follow algorithm.

| **Investigations** | **Potential results** |
| --- | --- |
| **❖ First line** |  |
| Dietary/lifestyle/medications assessment | High/ low fibre intake (use fibre quiz, <10 points low intake, >20 points high fibre intake)  High use of sorbitol - containing sugar chewing gum or sweets  High caffeine intake  High alcohol intake  High liquorish intake |
|  | Drug incuded: e.g  PPI’s  Laxatives  Beta blockers  Metformin |
| Blood test (TFTs, U&Es, Calcium, Albumin, CRP, B12, RBC Folate, glucose) | Abnormal results |
| Serum Mg2+ | Mg2+ low |
| Stool sample: for microscopy, culture and Clostridium Difficile toxin | Stool contains pathogens |
| Stool sample for faecal elastase (needs to be formed sample) | EPI |
| OGD with duodenal aspirate and biopsies and Glucose hydrogen (methane) breath test  NB OGD not recommended until 6 weeks post-upper GI surgery for testing for SIBO | SIBO |
| SeHCAT scan  (MUST have occasional or regular type 6 or 7 stool for this test- unless on high dose morphine or loperamide)  NB if patient had ilestomy test not indicated | BAM |
| Flexible sigmoidoscopy with biopsies from non-irrittated bowel  (avoid biopsies from areas obviously irradiated in sigmoid and rectum)  NB flexi sigmoidoscopy not recommended until 6 weeks post-lower GI surgery  For ano/rectal cancer undergoing chemo-radiation with grade 1 or 2 diarrhoea not to do flexi sigmoidoscopy until 6 weeks after completing treatment. If grade 3 diarrhoea ask oncology to consider | Macroscopic colitis  CMV |
| **❖ Second line** |  |
| Colonoscopy with biopsies (if Flexible Sigmoidoscopy normal and no other cause found) | Macroscopic or microscopic colitis  Organic cause (e.g. infection, inflammation, neoplastic) |
| **❖ Third line** |  |
| Fructose Breath Test | Fructose intolerance /malabsorption |
| **❖ Fourth line** |  |
| Sucrose Breath Test | Sucrose intolerance /malabsorption |

## Dysphagia – Solid food / Liquids / Saliva

Difficulty to swallow solids

| **Symptoms / Toxicity** | **Grade 0** | **Grade 1** | **Grade 2** | **Grade 3** |
| --- | --- | --- | --- | --- |
| **Dysphagia**  Duration? Assess patients urinary output & oral intake. | None | Symptomatic. Able to eat regular diet | Symptomatic. Altered eating. Reduced fluid intake. | Symptomatic and unable to eat diet and fluids. |
| **Action** | None | Monitor | Follow the Algorithm | Emergency  Inform the oncology team immediately |

If occurring occasionally in lower GI patients and bothersome, follow algorithm.

| **Investigations** | **Potential results** |
| --- | --- |
| Symptom history | Difficulty with liquids + solids |
|  | Neurological disorder:  - spasm  - sclerodema  - achalasia |
| If High dysphagia (back of throat or upper oesphagus):  Ask the oncology team to consider  Barium swallow or to arrange oesophagial ring | Inflammation (acid/ bile/ pepsin)  Local infection (viral/fungal) |
|  | Spasm |
|  | Oesophageal stricture |
| If Mid-lower oesophagus dysphagia:  OGD  NB OGD not recommended until 6 weeks post-upper GI surgery | Mechanical problem:  - lower oesophageal ring |
|  | Stricture |
|  | Inflammation (acid/ bile/ pepsin) |
|  | Spasm |
|  | Oesophageal stricture |
|  | Malignancy |
|  | Local infection (viral/fungal) |

## Early satiety

Feeling full after eating a small amount of food

NB if patient gastric tumour, only test for SIBO if worsening with chemotherapy/progressive. If had upper-GI resection wait 6 weeks post-operatively before following algorithm. If **total gastrectomy,** below not applicable.

| **Investigations** | **Potential results** |
| --- | --- |
| Symptom assessment | History of diabetes and high blood sugar levels  (gastric cancer) |
| Medication assessment | Anticholinergic drugs |
| Glucose Hydrogen Methane Breath Test  OGD + D2 Aspirate | SIBO |
|  | Malignancy / tumour recurrence |

## Faecal incontinence

Soiling / leakage / using pads

| **Investigations** | **Potential results** |
| --- | --- |
| Blood test (TFTs, U&Es, Calcium, Albumin, CRP, B12, RBC Folate, Mg2+) | Abnormal result |
| Flexible sigmoidoscopy  NB flexi sigmoidoscopy not recommended until 6 weeks post-lower GI surgery | Pelvic floor dysfunction and faecal incontinence/leakage |
|  | Anal sphincter defect |
|  | Mucosal prolapse |
| Abdominal X-ray  NB only do if regularly having symptom  (if had CT of abdomen in past month ask doctor to review this instead) | Bowel obstruction/ overflow |
|  | Constipation with overflow diarrhoea |
| In mucus leakage please see page 27 |  |

## Flatulence (rectal)

| **Investigations** | **Potential results** |
| --- | --- |
| **❖ First line** |  |
| Dietary assessment | Excess/deficient fibre intake  Inadequate fluids |
| OGD + D2 aspirate  Glucose, hydrogen,  Methane breath test.  NB OGD not recommended until 6 weeks post-upper GI surgery for testing for SIBO | SIBO |
| **❖ Second line** |  |
| Abdominal X-ray  (if had CT of abdomen in past month ask doctor to review this instead) | Faecal loading |

## Halitosis

An unpleasant odour emitted from the mouth

| **Investigations** | **Potential results** |
| --- | --- |
| **❖ First line** |  |
| Dietary assessment | Strong smelling food:  Onions, garlic, coffee |
| Medications assessment | Nitrates  Phenothiazines |
| History | Smoking |
| Visual inspection of mouth | Gum disease  Tooth decay |
|  | Candida infection |
|  | Xerostomia |
| **❖ Second line** |  |
| OGD + D2 Aspirate  NB OGD not recommended until 6 weeks post-upper GI surgery for testing for SIBO  Glucose, Hydrogen, Methane Breath Test | Gastric dysmotility |
|  | Ulceration |
|  | Duodenal obstruction |
|  | Malignancy / tumour recurrence |
|  | SIBO |

## Hiccups (singultus)

An involuntarily contraction of the diaphragm resulting in a hiccup sound being produced at the top of the windpipe

| **Investigations** | **Potential results** |
| --- | --- |
| **❖ First line** |  |
| Medications assessment | Corticosteroids  Benzodiazepines  Barbiturates  Opioids  Methyldopa |
| Blood test (U&Es, FBC, Ca^2+^, CRP) | Infection with vagus irritation:  Pleuritis  Pharyngitis  Meningitis |
|  | Metabolic:  Hypokalaemia  Hypercalcaemia |
| OGD + duodenal aspirate  NB OGD not recommended until 6 weeks post-upper GI surgery for testing for SIBO | GORD |
| Glucose, Hydrogen, Methane Breath Test | SIBO |
| **❖ Second line** |  |
| Ask oncology team/ supervising clinician re:  Chest X-ray  CT Chest & upper abdomen | Effusion/elevated diagram, mass on diagram, irritation of phrenic nerve |

## Ilestomy

NB for ilestomy patients type 6-7 stools are normal and nocturnal defecation normal, not for algorithm investigations for this.

For ilestomy patients must assess frequency of defecation by measuring output in jug over 24 hours prior to booking investigations. For ileostomy an output of >500ml in 24 hours is abnormal.

NB: If rectal discharge or bleeding, please refer to page 26 and 8.

| **Investigations** | **Potential results** |
| --- | --- |
| **❖ First line** |  |
| Glucose, Hydrogen, Methane Breath Test | SIBO |
| OGD+D2 Aspirate | SIBO |
| Faecal Elastase (if unable to obtain formed sample ask oncology team to consider trial of Creon). | EPI |
| **❖ Second line** |  |
| If >1000ml in 24 hours | Ask oncology team to consider a referral to gastroenterology. |

## Jaundice

**For immediate assessment**

**Inform oncology team**

## Loss of sensation

Unable to discriminate between need to defecate and pass urine

**For immediate assessment within 30 minutes**

**Inform oncology team**

## Mucus Discharge

| **Investigations** | **Potential results** |
| --- | --- |
| Fibre quiz | Excessive dietary fibre intake |
| Flexible sigmoidoscopy  NB If anal or rectal cancer to only do if a new symptom and has not had colonscopy/flexible sigmoidoscopy in the past three months, flexi sigmoidoscopy not recommended until 6 weeks post-lower GI surgery | Haemmorroids  Lesion: Anal/ Rectal |
|  | Anorectal ulcer |
|  | Neoplastic |
|  | Rectal mucosal prolapse |
|  | Traumatic ulceration / solitary rectal ulcer syndrome. |
|  | Radiation related  Carpet villous adenoma |
|  | New neoplasm |
|  | IBD |
|  | Diversion Colitis |
| OGD + D2 Aspirate  NB OGD not recommended until 6 weeks post-upper GI surgery for testing for SIBO | SIBO |
| Glucose, Hydrogen, Methane Breath Test | SIBO |

## Nausea and vomiting

**NB: If regurgitation, go to page 35.**

| **Symptoms / Toxicity** | **Grade 0** | **Grade 1** | **Grade 2** | **Grade 3** |
| --- | --- | --- | --- | --- |
| **Vomiting**  How many episodes / day? How many days?  Constipation or diarrhoea? (see specific toxicity)  Assess patient’s urinary output & oral intake. | None | 1 episode in 24 hours  Review anti emetics | 2-5 episodes in 24 hours  Review anti emetics according to Trust policy | ≥6 episodes in 24 hours |
| **Nausea**  Duration? What is the patient’s oral intake?  Is the patient taking anti emetics as prescribed?  Assess patient’s urinary output. | None | Able to eat/drink reasonable intake  Review anti emetics | Can eat/drink but intake significantly decreased  Review anti emetics according to Trust policy | No significant intake |
| **Action** | None | Monitor | Follow the Algorithm | Emergency  Inform the oncology team immediately |

**If grade 0-3 for <7 days monitor. If grade** 3 inform oncology team and CNS. If symptoms ≥7 days follow algorithm.

| **Investigations** | **Potential results** |
| --- | --- |
| **❖ First line** |  |
| Ask oncology team to consider Fundoscopy | Raised ICP |
| Blood test (FBC, U&Es, LFT, Ca2+, CRP, BGL)  Medication assessment | Metabolic abnormality |
|  | Liver / biliary abnormality |
|  | Suggestive of infection |
| Urine dip | Metabolic abnormality e.g. glucosuria, ketonuria |
|  | Infection |
| OGD + duodenal aspirate and  Glucose hydrogen methane breath test  NB OGD not recommended until 6 weeks post-upper GI surgery for testing for SIBO | Inflammatory / ulcerative disease |
|  | Gastric dysmotility |
|  | Bleeding oesophageal varices/ gastric ulcer |
|  | SIBO |
| **❖ Second line.** Ask oncology team to consider: | |
| CXR / CT / MRI (including CNS) | Biliary / hepatic / pancreatic aetiology  Local or distal infection |
|  | Raised intra-abdominal pressure e.g. ascites |
|  | Central nervous system pathology |
| Blood test (Morning Cortisol) | Abnormal result |

## Pain (Upper Abdominal/Epigastric pain)

| **1** | **2** | **3** | **4** | **5** | **6** | **7** | **8** | **9** | **10** |
| --- | --- | --- | --- | --- | --- | --- | --- | --- | --- |
| **No pain** | **Mild pain** | | **Moderate pain** | | **Severe pain** | | **Very severe pain** | | **Worst pain** |
| **Follow algorithm** | | | | | **Inform Oncology Doctor same day and then follow algorithm.** | | | | |

If acute pain or severe-worse pain emergency referral to oncology team

If chronic pain, to follow algorithm

| **Investigations** | **Potential results** |
| --- | --- |
| **❖ First line** |  |
| Blood test (FBC, U&Es, LFT, Calcium, CRP, B12, RBC Folate, amylase) | Abnormal results  Pancreatitis |
| OGD + duodenal aspirate and  Glucose hydrogen methane breath test  NB OGD not recommended until 6 weeks post-upper GI surgery for testing for SIBO | Inflammation |
|  | Local infection (viral / fungal) |
|  | Stricture |
|  | Spasm |
|  | Malignancy / tumour recurrence |
|  | SIBO |
| Abdominal X-ray  NB only do if currently experience symptom, not to do for intermittent pain  (if had CT of abdomen in past month ask doctor to review this instead) | Bowel obstruction  Severe faecal loading |
| **❖ Second line. Ask the oncology team to consider:** | |
| US/CT abdo | Biliary tree obstruction, gallstones, |
|  | Pancreatic duct problems, |
|  | Renal stones |
|  | Ascities |
|  | Ischaemia |
|  | Recurrence |
| Chest Xray | Infection |
| ECG/ Exercise Test | Cardiac ischaemia |

## Pain (Lower abdominal)

| **1** | **2** | **3** | **4** | **5** | **6** | **7** | **8** | **9** | **10** |
| --- | --- | --- | --- | --- | --- | --- | --- | --- | --- |
| **No pain** | **Mild pain** | | **Moderate pain** | | **Severe pain** | | **Very severe pain** | | **Worst pain** |

http://www.npc.nhs.uk/therapeutics/pain/overview/resources/pda_pain_overview.doc

If sudden onset/acute inform oncology immediately.

If chronic and symptoms >1 week follow algorithm

| **Investigations** | **Potential results** |
| --- | --- |
| **❖ First line** |  |
| Diet assessment and Fibre quiz | Inappropriate fluid and fibre intake  Excessive sorbitol  Excessive caffeine |
| Medication assessment | Drug induced: eg.   - Opioid - ondansetron - anti-muscarinics - loperamide - iron supplement - statin - metformin |
| Blood test ( FBC, U&Es, LFTS, Calcium, ESR, CRP) | Abnormal results |
| Abdominal X-Ray  NB only do if currently experience symptom.  (if had CT of abdomen in past month ask doctor to review this instead) | Faecal loading / faecal impaction |
| OGD and duodenal aspirate  and glucose, hydrogen, methane  breath test.  NB OGD not recommended until 6 weeks post-upper GI surgery for testing for SIBO | SIBO |
|  | Newly diagnosed IBD |
| **❖ Second line. Ask the oncology team to consider:** | |
| Ultrasound/CT of abdo and pelvis | Suggestive of gallstones, IBD, tumour recurrence other |

## Pain (anal / perianal / rectal): related to defaecation

| **1** | **2** | **3** | **4** | **5** | **6** | **7** | **8** | **9** | **10** |
| --- | --- | --- | --- | --- | --- | --- | --- | --- | --- |
| **No pain** | **Mild pain** | | **Moderate pain** | | **Severe pain** | | **Very severe pain** | | **Worst pain** |

If sudden onset/acute inform oncology immediately.

If chronic and symptoms >1 week follow algorithm

| **Investigations** | **Potential results** |
| --- | --- |
| **❖ First line** |  |
| Medication assessment | On Nicorandil |
| Ask oncology doctor to do visual assessment and gentle palpitations | Haemorrhoids |
|  | Anal fissure |
|  | Abscess |
| **❖ Second line** |  |
| Flexisig  NB flexi sigmoidoscopy not recommended until 6 weeks post-lower GI surgery | Anal fissure |
| **❖ Third line. Ask oncology team to consider:** | |
| MRI | Anorectal fistula |
|  | Anorectal ulcer |
|  | Muosal propalpse/Solitary rectal ulcer |
|  | Neoplastic ulcer |
|  | Radiation related |
|  | Abscess |

## Pain (anal / perianal / rectal): not related to defecation)

| **Investigations** | **Potential results** |
| --- | --- |
| **❖ First line** |  |
| Flexible sigmoidoscopy | Anal fissure |
|  | Proctalgia Fugax |
|  | Anorectal ulcer |
|  | Recurrent cancer |
|  | Colitis |
|  | Proctitis |
| **❖ Second line** |  |
| Ask oncology team to consider MRI and CT | Abscess |

## Pruritus (perianal)

(itchiness)

NB below investigations not recommend until 6 weeks after completion of radiotherapy

| **Investigations** | **Potential results** |
| --- | --- |
| **❖ First line** |  |
| Symptom assessment | Perianal pruritus mainly present during the night due to excess pancreatic enzyme replacement |
| Ask oncology team to do visual assessment | Abnormalities  If present ask oncology team re dermatology referral |
| Flexible sigmoidoscopy  NB flexi sigmoidoscopy not recommended until 6 weeks post-lower GI surgery | Haemmorroids |
|  | Anal fissure |
|  | Anorectal abcess |
|  | Anorectal ulcer |
|  | Mucosal prolapse/ solitary rectal ulcer |
|  | Neoplastic ulcer |

## Reduced appetite

| **Investigations** | **Potential results** |
| --- | --- |
| **❖ First line** |  |
| History assessment | Depression |
|  | Anxiety |
|  | Underlying eating disorder |
|  | Pre-existing co-morbidities:   - Cardiac failure - COPD - Chronic kidney disease - Chronic liver disease |

## Reflux (Acid / Bile) / heartburn

| **Investigations** | **Potential results** |
| --- | --- |
| **❖ First line** |  |
| Symptom assessment | Inflammation |
| Glucose, Hydrogen and methane Breath test  OGD and D2 aspirate  NB OGD not recommended until 6 weeks post-upper GI surgery for testing for SIBO | SIBO |
|  | Inflammation / ulceration |
|  | Bile reflux |
|  | Infection eg Fungal/ viral |
|  | Malignancy / tumour recurrence |
| **❖ Second line. Ask the oncology team to consider:** | |
| Barium swallow | Oesophageal spasm |
| ECG / exercise test | Cardiac related |

## Regurgitation

The expulsion of material from the mouth, pharynx, or oesophagus, usually characterized by the presence of undigested food and symptoms are worse post chemotherapy.

| **Investigations** | **Potential results** |
| --- | --- |
| **❖ First line** |  |
| OGD and D2 aspirate  Breath test | Mechanical problem:  Lower oesophageal ring/stricture/pharyngeal pouch |
|  | Inflammation |
|  | Malignancy / tumour recurrence |
|  | Small bacteria overgrowth |
| **❖ Second line. Ask the oncology team to consider:** | |
| CT chest, abdo, pelvis | Raised intra-abdominal pressure e.g. ascites |

## Steatorrhoea

(the presence of excess fat in the stool. Symptoms include seeing oil, pale/grey stool, stool floats, hard to flush, splatters, offensive smell. To follow algorithm even with intermittent symptoms)

| **Investigations** | **Potential results** |
| --- | --- |
| **❖ First line** |  |
| Stool sample for faecal elastase | EPI |
| Blood test (coeliac screen (if not done at baseline), TFT, U&Es, B12, RBC Folate), CRP  For Addison’s disease:  1st check sodium, potassium and urea.  2nd If low sodium, +/- high potassium +/- high urea then check morning cortisol  3rd If abnormal then order synacthen test. | Addison’s disease |
|  | Coeliac disease |
|  | Thyroid dysfunction |
| SeHCAT scan  Must have type 6 or 7 stool  NB if patient had ilestomy test not indicated |  |
|  | Intestinal parasites |
| OGD+D2 aspirate  Glucose, hydrogen, methane breath test  NB OGD not recommended until 6 weeks post upper- GI surgery for testing for SIBO | SIBO |
| **❖ Second line. Ask the oncology team to consider:** |  |
| CT abdo pelvis | Small intestinal disease other than radiotherapy induces  (e.g lymphoma) |

## Tenesmus

(a feeling of constantly needing to pass stools, despite an empty rectum)

NB if rectal/colon cancer only to do flexible sigmoidoscopy 6 weeks after completing radiotherapy

| **Investigations** | **Potential results** |
| --- | --- |
| **❖ First line** |  |
| Flexible sigmoidoscopy  NB flexi sigmoidoscopy not recommended until 6 weeks post-lower GI surgery | Radiation proctopathy |
|  | Anterior resection syndrome |
|  | Significant polyp |
|  | Infection |

## Weight loss (unintentional)

Significant weight loss ≥10% in past 3-6 months

| **Investigations** | **Potential results** |
| --- | --- |
| **❖ First line** |  |
| Dietary assessment | Inadequate dietary intake |
| Blood tests (TFT, CRP, morning cortisol, sodium, potassium and urea)  For Addison’s disease:  1st check sodium, potassium and urea.  If low sodium, high potassium ± high urea then measure cortisol. If this is deranged then order synacthen test. | Abnormal results e.g.   - Thyrotoxicosis - New onset diabetes mellitus - Addison’s disease |
| Faecal Elastase | Pancreatic Insufficiency |
| Ask the oncology team to consider:  PET / CT chest abdomen pelvis | Infection |
|  | Inflammation |
|  | Malignancy / tumour recurrence |
