# Supplementary material for: The FOCCUS study: a prospective evaluation of the frequency, severity and treatable causes of gastrointestinal symptoms during and after chemotherapy
Source: Support Care Cancer. 2020 Jul 16;29(3):1443–53. doi: 10.1007/s00520-020-05610-x (PMC7843552; doi:10.1007/s00520-020-05610-x)
Supplement: Supplementary file 2 — (DOCX 237 kb) [file 520_2020_5610_MOESM2_ESM.docx]

**Figure 1 supplement: The GSRS questionnaire which patients completed at every assessment**

**
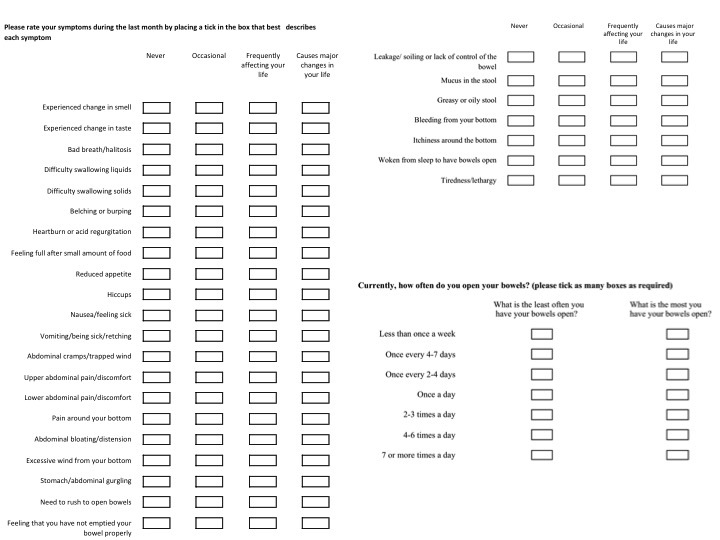
**

**Figure 2 supplement: The percentage of patients who received chemotherapy, the percentage who required dose reduction and the percentage who stopped one or more of the initially prescribed chemotherapy medications each month through the study.**

**Figure 3 supplement: Stool type according to the Bristol stool chart at any time during the study**

**(patients may have reported more than one type over each reporting period)**

**Figure 4a supplement: The GSRS questionnaire asked patients to state the least number of times they opened their bowels**

**Figure 4b supplement: the most number of times they opened their bowels since they last completed the questionnaire.**

**Figure 5 supplement: Percentage of patients with varying degrees of faecal continence as measured using the St Marks Score over time (a score of zero suggests perfect continence and a score of 24 suggest the worst possible faecal incontinence).**

**Percentage of patients**

**Figure 6 supplement: FACT G Quality of life scores (mean with standard deviations indicated) for each of the four domains, physical, social, emotional and functional.**

**Figure 7 supplement: Number of endoscopic procedures offered to patients to help with their GI symptoms, the numbers arranged because the patient initially agreed to have them done and the number actually performed**

**Number of procedures**

**Figure 8a supplement: 7 day retention results of the 23-seleno-25-homotaurocholic acid (SeHCAT) scan: It was offered on 181 occasions to participants and 30 patients accepted to undergo this test. The number of people in each category of severity of bile acid malabsorption is indicated.**

**Figure 8b supplement: Time when the SeHCAT scan was offered and the number taken up and the number of those which were positive.**
